# Supplementary material for: MAPLE (modular automated platform for large-scale experiments), a robot for integrated organism-handling and phenotyping
Source: eLife. 2018 Aug 17;7:e37166. doi: 10.7554/eLife.37166 (PMC6193762; doi:10.7554/eLife.37166)
Supplement: Supplementary file 1. — Spreadsheet listing MAPLE’s components, parts numbers, vendor links, and fabrication techniques. [file elife-37166-supp1.pdf]

MAPLE Fly-Handling Robot Bill of Materials

Last updated: 12/3/2017

Color code:		
Off-the-shelf	3d-printed	Waterjet
Customized	Laser cut	Machined
Alt. source		

	Qty	Price	Ext. Price	Source	Part #	X/Y only	LLT?	Notes	Alternate source
**Structural**									
40mm sq x 1120mm Extrusion	2	$29.90	$59.80	http://us.misumi-ec.c	HFSB5-4040-1120	*			
20mm x 40mm x 410mm Extrusion	4	$6.84	$27.36	http://us.misumi-ec.c	HFSB5-2040-410	*			
20mm x 40mm x 370mm Extrusion	2	$6.17	$12.34	http://us.misumi-ec.c	HFSB5-2040-370	*			
20mm x 40mm x 1120mm Extrusion	2	$18.70	$37.40	http://us.misumi-ec.c	HFSB5-2040-1120	*			
20mm sq x 370mm Extrusion	2	$3.25	$6.50	http://us.misumi-ec.c	HFSB5-2020-370	*			
Wide corner bracket	8	$3.38	$27.04	http://us.misumi-ec.c	HBLFSDK5	*			
Narrow corner bracket	8	$2.85	$22.80	http://us.misumi-ec.c	HBLFSSW5	*			
Sheet metal brace	8	$10.50	$84.00	http://us.misumi-ec.c	SHPTCUL5-SET	*			
Spacer stick	1	$4.00	$4.00	http://tapplastics.con	1/4" thick acrylic sheet, cut from 8" x 2.75"	*			
Laser cutting		varies							
End panel	1	$8.00	$8.00	http://tapplastics.con	1/4" thick acrylic sheet, cut from 8" x 16"	*			
Laser cutting		varies				*			
Work surface	1	$50.00	$50.00	http://tapplastics.con	1/4" thick acrylic sheet, cut from 16.5" x 46"				
Laser cutting		varies							
		**Subtotal:**	$339.24						
**Motion**									
Motor bracket	2	-	$9.19	OnlineMetals.com	6061-T6 Al, 1/4" thick - 8" x 8"	*			
Waterjet cutting / machining		varies				*			eMachineShop.com
Idler pulley bracket	2	-		OnlineMetals.com	6061-T6 Al, 1/4" thick - included w/above	*			
Waterjet cutting / machining		varies				*			eMachineShop.com
NEMA 17 stepper	2	$11.50	$23.00	RobotDigg.com	MNEMA17-60	*	Y		https://www.amazon.com/Torque-Current-Stepper-Motor-Extruder/dp/B00PNEQP1G/
X axis rail	2	$81.30	$162.60	IGUS.com	AWUM-12 @ 1116mm length	*			
X axis bearing	4	$51.93	$207.72	IGUS.com	OJUM-06-12	*			
Driling & tapping						*			
Y axis	1	$49.37	$49.37	IGUS.com	WS-10-120 @ 453mm length	*			
Driling & tapping						*			
Y upper plate	1	-	$34.56	OnlineMetals.com	6061-T6 Al, 1/8" thick - 12" x 18"	*			
Waterjet cutting / machining		varies				*			eMachineShop.com
Y lower plate	1	-	$12.16	OnlineMetals.com	6061-T6 Al, 3/16" thick - 8" x 8"	*			
Waterjet cutting / machining		varies				*			eMachineShop.com
Y bearings	4	$5.25	$21.00	IGUS.com	WJ200UM-01-10	*			
Belt clamp	2		$0.00	3d printed		*			Shapeways.com
Belt tensioner	2		$0.00	3d printed		*			Shapeways.com
X axis limit switch holder	1		$0.00	3d printed		*			Shapeways.com
Pulley, motor	2	$1.80	$3.60	RobotDigg.com	GT2-20T-5B-6	*	Y		https://www.amazon.com/Qunqi-Aluminum-Timing-Pulley-Printer/dp/B01IMR6OR0/
Pulley, idler	8	$1.80	$14.40	RobotDigg.com	GT2-20T-8B-6	*	Y		2x https://www.amazon.com/CycleMore-Aluminum-Pulleys-Printer-Reprap/dp/B016ZRJGBK/
Idler bearing	8	$2.00	$16.00	McMaster.com	6679K11	*			
Z axis plate	1	-		OnlineMetals.com	6061-T6 Al, 1/8" thick - included w/above				
Waterjet cutting / machining		varies							eMachineShop.com
T brackets	1	-	$20.16	OnlineMetals.com	6061-T6 Al, 3/8" thick - 6" x 12"				
Waterjet cutting / machining		varies							eMachineShop.com
Drag chain plate	1	-		OnlineMetals.com	6061-T6 Al, 1/8" thick - included w/above	*			
Waterjet cutting / machining		varies				*			eMachineShop.com
X axis drag chain	6	$16.05	$96.30	McMaster.com	4516T48 (6' total)	*			
Y axis drag chain	2	$12.47	$24.94	McMaster.com	4516T46 (2' total)	*			
Y axis drag chain mount	1		$0.00			*			Shapeways.com
GT2 belt	1	$15.00	$15.00	RobotDigg.com	GT2-6-OPEN_10Meters	*	Y		2x https://www.amazon.com/Meters-pitch-wide-Timing-printer/dp/B00F2IQNX8/
Z slides	3	$317.20	$951.60	IGUS.com	SLN-D740679-2		Y		
Z mount	3	-		OnlineMetals.com	6061-T6 Al, 1/8" thick - included w/above				
Waterjet cutting / machining		varies							eMachineShop.com
4mm spacer	10	$1.25	$12.50	McMaster.com	93657A404	*			
2mm spacer	6	$1.36	$8.16	McMaster.com	93657A402	*			
1/4" spacer	4	$8.72	$34.88	McMaster.com	94639A569	*			
Shoulder bolt, Y carriage, 25mm	4	$5.82	$23.28	McMaster.com	90265A145	*			
Shoulder bolt, idler pulleys	4	$5.49	$21.96	McMaster.com	90265A144	*			
		**Subtotal:**	$1,762.38						
**Manipulation**									
Manifold block	2	15.16	$30.32	Clippard.com	M-E10M-02				
Solenoid valve	4	$24.68	$98.72	Clippard.com	E210C-2W012				
Tubing	50	$0.21	$10.50	McMaster.com	50315K68 - 25' red, 25' blue				
Tube fittings	10	$2.58	$25.80	McMaster.com	7880T389 - Nickel-plated brass				
Object manipulator manifold	1	varies		Machined					eMachineShop.com or 3d printed
Organism manipulator manifold	1	varies		Machined					eMachineShop.com or 3d printed
Vacuum cup	1	$6.25	$6.25	McMaster.com	5427A628 - 1/8 NPT, Male				
Luer lock fitting	1	$5.24	$5.24	McMaster.com	51525K23 - Impact resistant				
Organism manipulator switch holder	1		$0.00	3d printed					Shapeways.com
Fly needle	1	$3.41	$3.41	McMaster.com	6710A61				
0.042" dispenser needle	1	$12.18	$12.18	McMaster.com	75165A553				
0.028" dispenser needle	1	$12.15	$12.15	McMaster.com	75165A682				
		**Subtotal:**	$204.57						
**Electronics**									
SmoothieBoard	1	$162.97	$162.97	https://shop.uberclo	Smoothieboard 5x-xM	*			
Smoothie USB cable	1	$4.99	$4.99	Amazon.com	B00NH11KIK	*			
Camera USB cable	1	$9.99	$9.99	Amazon.com	B00HNF0OS8				

MAPLE Fly-Handling Robot Bill of Materials

Last updated: 12/3/2017

**Color code:**		
Off-the-shelf	3d-printed	Waterjet
Customized	Laser cut	Machined
Alt. source		

	Qty	Price	Ext. Price	Source	Part #	X/Y only	LLT?	Notes	Alternate source
Ribbon breakout PCB	1	$29.15	$29.15	OSHPark.com	https://oshpark.com/shared_projects/rHsVOk			These two PCBs are custom, but can be ordered from OSHPark.	
Smoothieboard ribbon PCB	1	$109.00	$109.00	OSHPark.com	https://oshpark.com/shared_projects/Tl6vECx			Unfortunately, OSHPark.com will only build PCBs in quantities that are multiples of 3.	
Electronics components	see next sheet		$111.62						
		**Subtotal:**	$427.72						
**Vision**									
Camera mount	1		$0.00	3d printed					Shapeways.com
Camera	1	$359.00	$359.00	TheImagingSource.c	DFM 72BUC02-ML		Y		
Lens holder	1	$16.80	$16.80	TheImagingSource.c	TLH 10-2				
Lens	1	$28.00	$28.00	TheImagingSource.c	TBL 9.6-2 C 3MP				
LED ring	1	$9.80	$9.80	Amazon.com	B00MJSJFPW				
		**Subtotal:**	$413.60						
**Fasteners & Misc. HW**									
Nuts for extrusion	2	$19.32	$38.64	http://us.misumi-ec.c	HNKK5-5	*			
Post-assy extrusion nuts, M5	1	$29.70	$29.70	http://us.misumi-ec.c	PACK-HNTASN5-5	*			
M6 x 16 round head hex screw	1	$5.57	$5.57	McMaster.com	92095A238	*			
M5 x 10 SHCS	2	$8.15	$16.30	McMaster.com	91292A124	*			
M5 x 6 SHCS	2	$4.63	$9.26	McMaster.com	91292A189	*			
M5 x 20 SHCS	4								
M5 x 25 shoulder bolt	4	$5.53	$22.12	McMaster.com	90265A145	*			
M5 x 20 shoulder bolt	4	$5.22	$20.88	McMaster.com	90269A144	*			
M3 x 25 SHCS	1	$6.40	$6.40	McMaster.com	91292A020	*			
M3 x 20 mm threaded rod	1	$7.33	$7.33	McMaster.com	93805A631				
M3 x 20 SHCS	1	$6.00	$6.00	McMaster.com	91292A123	*			
M3 x 14 low profile screw	2	$5.80	$11.60	McMaster.com	92095A168	*			
M3 x 12 SHCS	1	$4.40	$4.40	McMaster.com	91292A114	*			
M3 x 8 SHCS	1	$4.00	$4.00	McMaster.com	91292A112	*			
M3 washer	1	$1.62	$1.62	McMaster.com	93475A210	*			
M3 nut	1	$5.55	$5.55	McMaster.com	91828A211	*			
M2 x 8mm pan head screw	1	$4.52	$4.52	McMaster.com	92000A015				
M2.63 x 12mm for plastics	1	$14.33	$14.33	McMaster.com	99397A356	*			
M1.91 x 12mm for plastics	1	11.66	$11.66	McMaster.com	99397A077				
Zip tie mount	1	8.53	$8.53	McMaster.com	7582K11				
Spacer for camera	1	7.77	$7.77	McMaster.com	94639A464				
M2.5 x 10mm SHCS	1	4.94	$4.94	McMaster.com	91292A014				
M3 x 12 mm Shoulder screw	2	2.66	$5.32	McMaster.com	90265A122				
M3 spacers for PCBs	10	0.48	$4.80	McMaster.com	95947A008				
M5 flat washer	1	2.57	$2.57	McMaster.com	93475A240				
M5 nyloc nut	1	5.98	$5.98	McMaster.com	93625A200				
		**Subtotal:**	$259.79						
**Tools & Consumables**									
Epoxy	1	$54.96	$54.96	McMaster.com	75045A65	*			
M5 x 0.8 tap	1	$6.67	$6.67	McMaster.com	8305A16	*			
1/4-28 tap	1	5.35	$5.35	McMaster.com	2521A681				
M3 x 0.5 tap	1	8.09	$8.09	McMaster.com	8305A12				
M2.5 x 0.45 tap	1	9.59	$9.59	McMaster.com	8305A11				
Letter O drill bit	1	3.84	$3.84	McMaster.com	30595A46	*			
		**Subtotal:**	$88.50						
	**Total Parts Cost:**		**$3,495.80**						
