# Supplementary material for: MAPLE (modular automated platform for large-scale experiments), a robot for integrated organism-handling and phenotyping
Source: eLife. 2018 Aug 17;7:e37166. doi: 10.7554/eLife.37166 (PMC6193762; doi:10.7554/eLife.37166)
Supplement: Supplementary file 2. — Document outlining the steps to assemble MAPLE from parts, including frame, rails, effectors, control electronics, wiring and plumbing. Some maintenance information is included, as well as software instructions to test MAPLE’s basic functionality and communication protocols. [file elife-37166-supp2.pdf]

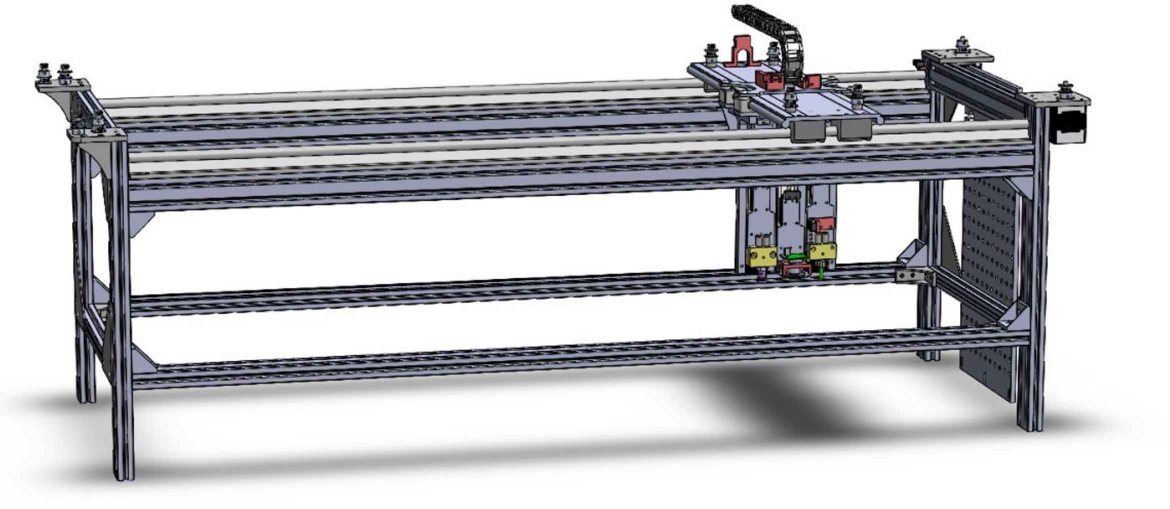

# MAPLE Build Instructions

Dave Zucker, FlySorter LLC

## Change Log

2016-12-15 - Version 2.0 - Initial build instructions

2017-07-27 - Version 2.1 - Doc updates (no design changes)

2017-11-25 - Version 2.2 - More details (no design changes)

2018-07-01 - Version 2.3 - Added cleaning instructions.

2018-09-05 - Version 2.4 - Added end plate and work surface instructions.

MAPLE Build Instructions
- Change Log
- Notes
- Documentation Sources & License
- Pre-Assembly
  - Aluminum Extrusions
  - 3D Printed Parts
  - Silicone Rubber Boot
  - Machined Aluminum Parts
  - Waterjet Aluminum Parts
    - Secondary operations:
  - Laser Cut Parts
  - X Axis Rails
  - X Axis Bearings
  - Y Axis
  - PCB Assembly
    - Smoothieboard
    - Smoothieboard-Ribbon PCB
    - Ribbon Breakout PCB
- Frame Assembly
- Y Axis Assembly
  - Rail Maintenance
- Assembling the Z Axes
  - Z0 - Object Manipulator
  - Z1 - Camera
  - Z2 - Organism Manipulator
    - Organism Manipulator Maintenance
  - Complete Z Assembly
- Wiring & Tubing
  - Threading the Drag Chains
    - Large Drag Chain
    - Small Drag Chain
  - Power Supply
  - Smoothieboard
  - Z Assembly

Air & Vacuum Tubing
Firmware
Alignment & Belt Tension
Testing

# Notes

These instructions will guide you through assembly of the MAPLE fly handling robot.

Before you begin, please note:

- Before you order parts or start 3D printing, read through this document thoroughly and make sure you understand what's required.
- We assume familiarity with and/or access to a machine shop, hand tools, 3D printer, soldering station, and more. If you don't have access to a 3D printer, there are online services that can provide parts, such as Shapeways.com, 3dhubs, and more. Similarly, eMachineShop.com will fabricate parts from CAD. But with that said, you will have to get your hands dirty at some point during this build, so it may not be for everyone.
- While we have made every effort to ensure the documentation is complete, we cannot be responsible for errors (though of course we will gladly try to help).
- The bill of materials for the MAPLE robot can be found here (https://docs.google.com/spreadsheets/d/1WS5JZ8TgN1G7_WdbVoJIBsS1zV8HpRZNDN9ckr8W_F8/edit?usp=sharing).
- Unfortunately, many of the parts (particularly fasteners) are sold in packs of 25, 50 or more, even if the assembly only uses a few. At this point, neither FlySorter nor the de Bivort Lab sells a ready-made kit of parts. The costs shown in the BOM do reflect purchasing full packs.
- Some fasteners are omitted from the CAD file. Their locations are indicated in this document.

# Documentation Sources & License

We have made the original CAD files (SOLIDWORKS 2016), STL files for 3D printing, and PDF drawings of all mechanical parts available on Github: https://github.com/FlySorterLLC/MAPLEHardware

If you do not have a license for SOLIDWORKS, you can still open the files using the free eDrawings Viewer (http://www.edrawingsviewer.com/).

The circuit boards in this project were designed using KiCAD EDA open source software (http://kicad-pcb.org/). The original design files are also in the Github repository.

All files are distributed under the GNU GPL v2 (see LICENSE file).

# Pre-Assembly

## Aluminum Extrusions

Tap the holes in one end of each of the four 410 mm long 20x40 extrusions with M5 threads:

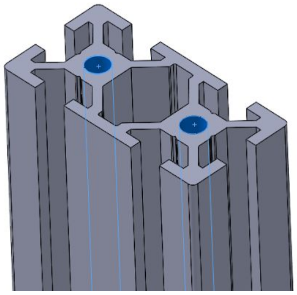

## 3D Printed Parts

Settings:

- Maximum 0.2 or (preferred) 0.15 layer thickness
- Minimum 0.6 mm top and bottom thickness
- Minimum 0.8 mm shell thickness
- Minimum 25% infill

Print the following (STL files available in Github):

| Part | Quantity |
|---|---|
| BeltClamp-AD-FU | 1 |
| BeltClamp-AU-FD | 1 |
| BeltAdjuster | 2 |
| DragChain-YMount | 1 |
| LimitSwitch-Holder-XAxis | 1 |
| Z2-Carrier-Switch | 1 |
| IS-Camera-Holder | 1 |
| Bellows-cavity-half | 2 |
| Bellows-core | 1 |
| 2mm & 4mm spacer | As needed |

Use a 3.2 mm drill to open these holes to the proper diameter:

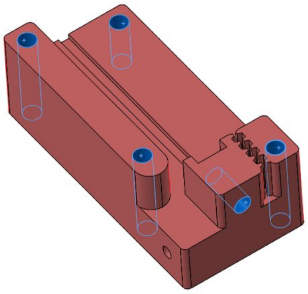

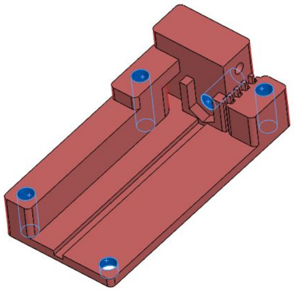

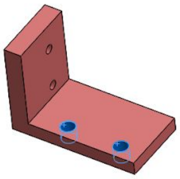

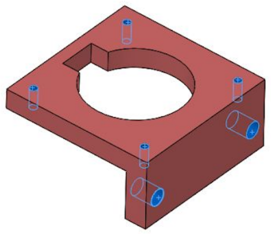

5.5mm drill:

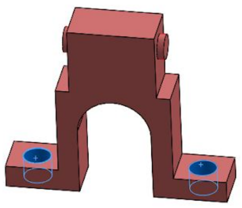

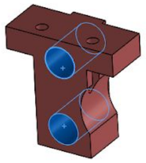

## Silicone Rubber Boot

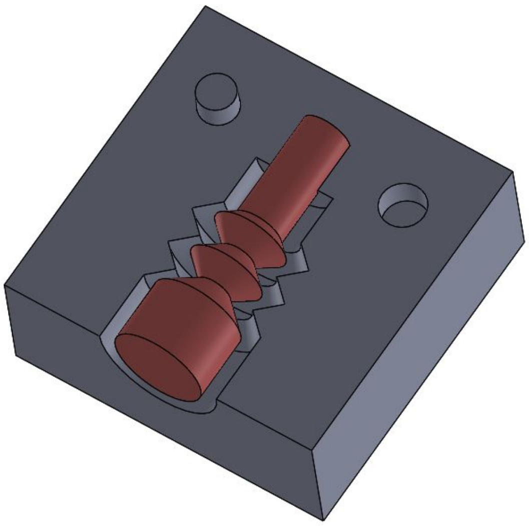

Coat the working faces of the three mold parts (two cavity halves and one core) in mold release (or vaseline, in a pinch). Clamp the two mold halves together, and fill the resulting cavity with liquid silicone (or some other elastomer), insert the core, and allow to cure. Trim off any excess, and remove from the mold.

## Machined Aluminum Parts

See PDF drawings for the organism and object manipulator manifolds in Github. It is also possible to 3D print these parts instead.

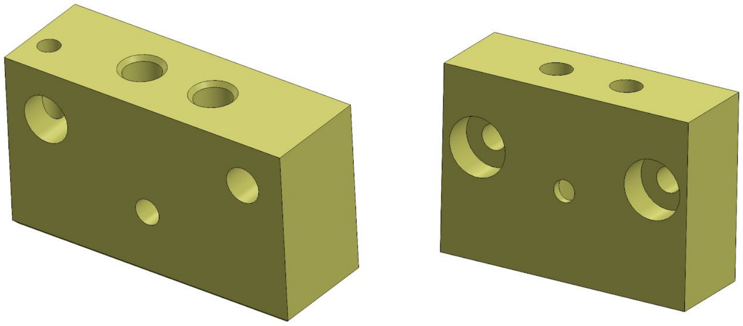

## Waterjet Aluminum Parts

See the DXF in the Drawings directory of the Github repository.

| Material | Part | Quantity |
|---|---|---|
| ⅛” Aluminum plate | Upper-Y-Plate | 1 |
| | DragChain-Plate | 1 |
| | Z-Mount | 3 |
| | Z-Plate | 1 |
| 3/16” Al plate | Lower-Y-Plate | 1 |
| ¼” Al plate | Stepper-Plate | 2 |
| | Idler-Plate | 2 |

If your waterjet parts are spot on, you should be able to tap holes without pre-drilling. Measure a couple and compare against the PDF/DXF to be sure.

### Secondary operations:

Tap M5 threads (drill to 4.2 mm if holes are small):

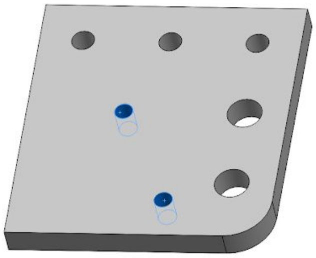

Tap M3 threads (drill to 2.5 mm if necessary):

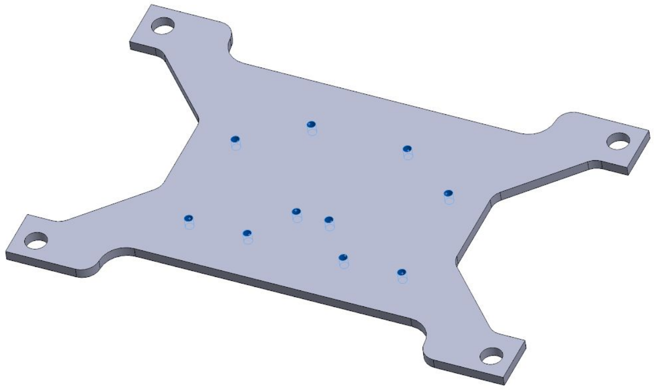

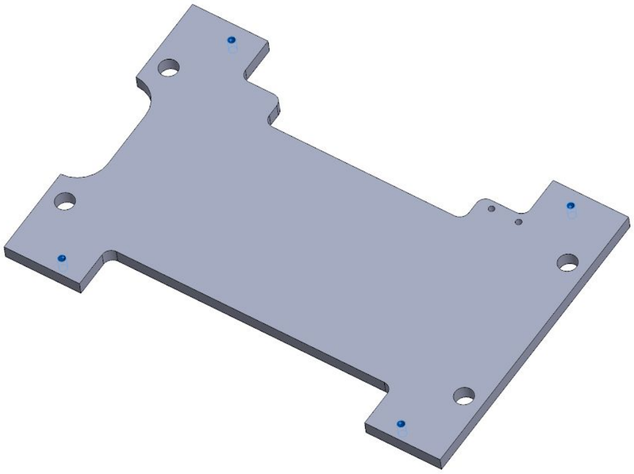

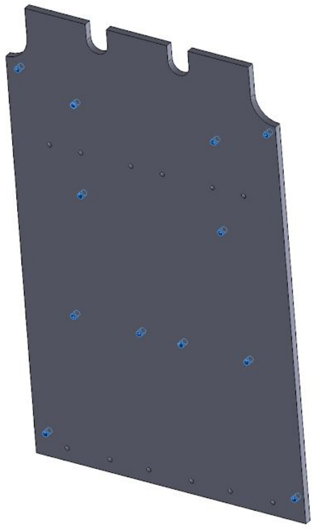

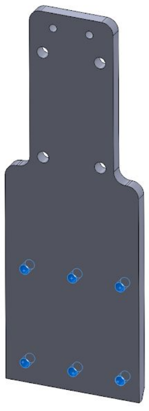

Tap M2.5 threads (drill to 2 mm if necessary):

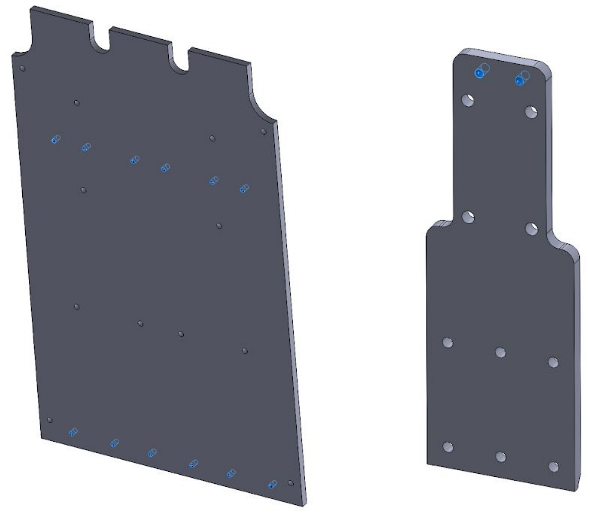

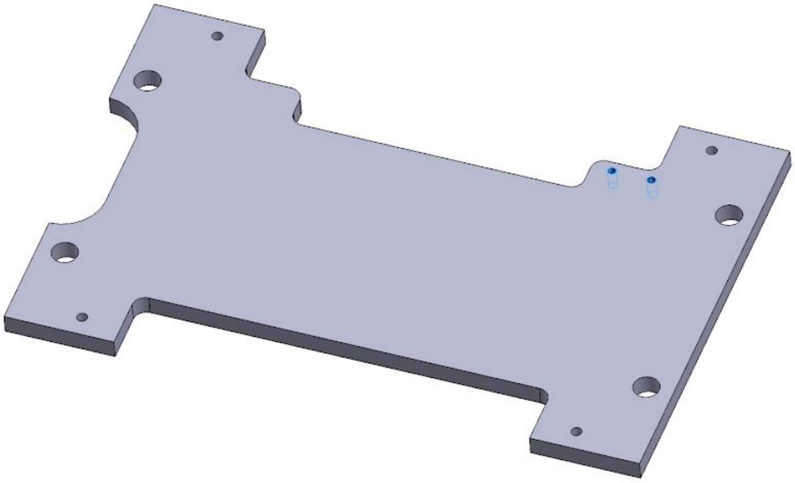

File / grind ~30° bevel:

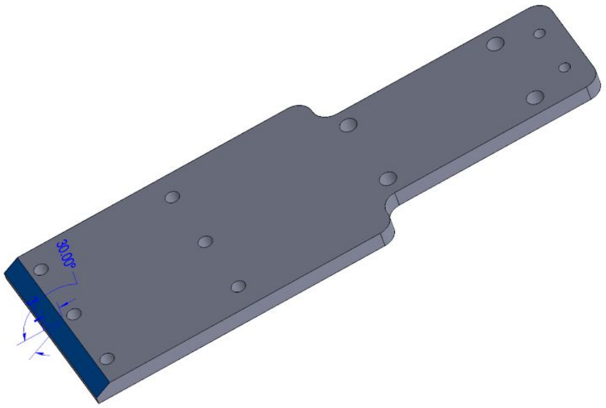


## Laser Cut Parts

Cut the spacer stick from ¼” or ⅛” acrylic:

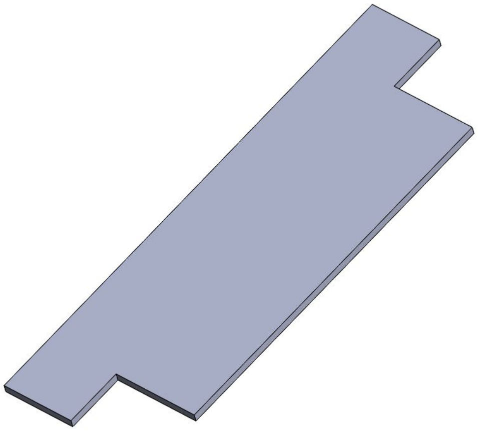

## X Axis Rails

After cleaning & degreasing the parts, glue 1116 mm long IGUS rails to the 1120 mm long 40mm square aluminum extrusions using epoxy. This flexible epoxy from McMaster is recommended (part number 75045A65), as it bonds well to anodized aluminum. Other glues may perform well, too. Use masking (or other non-gummy) tape to clamp the rails during glue up.

The width of the base of the supported shaft matches the 40mm Misumi extrusions. Make sure they are lined up during assembly (you can clamp flat parts on either side to ensure alignment).

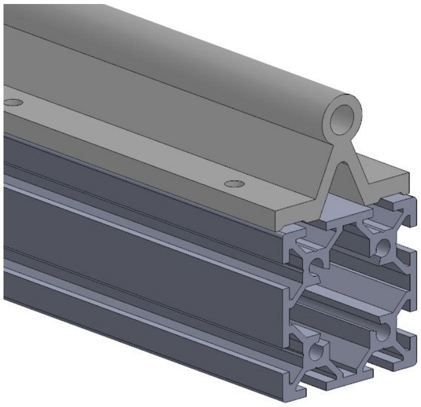

Allow glue to cure fully before assembling the frame.

## X Axis Bearings

Drill out & tap (M5) per drawing.

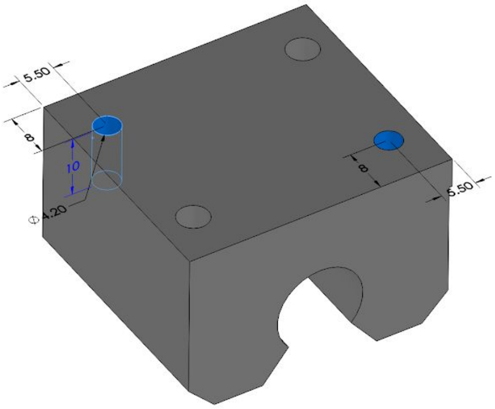


For two of these bearings, drill and tap a 10 mm deep hole on the side with the lip. Make a left and a right version by measuring from different ends for the two blocks.

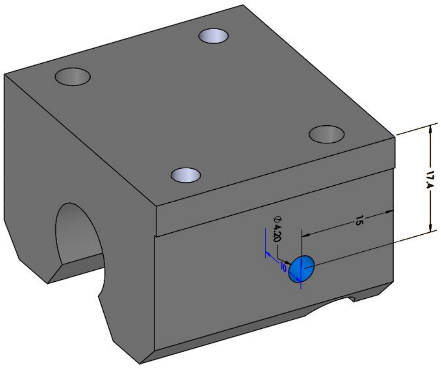


## Y Axis

Drill / mill holes in WS-10-120 part as shown on the PDF drawing:

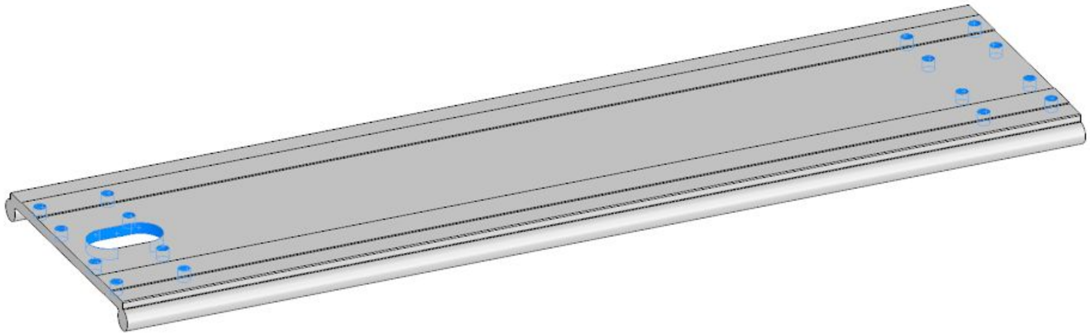

The slot (on the left-hand side of the part in the image above) is for wires and tubes to pass through. Drilling holes and sawing the waste in between them is perfectly acceptable here (if not the most aesthetically appealing), as is using a milling machine, and regardless of how you form that feature, be sure to deburr the edges.

# PCB Assembly

## Smoothieboard

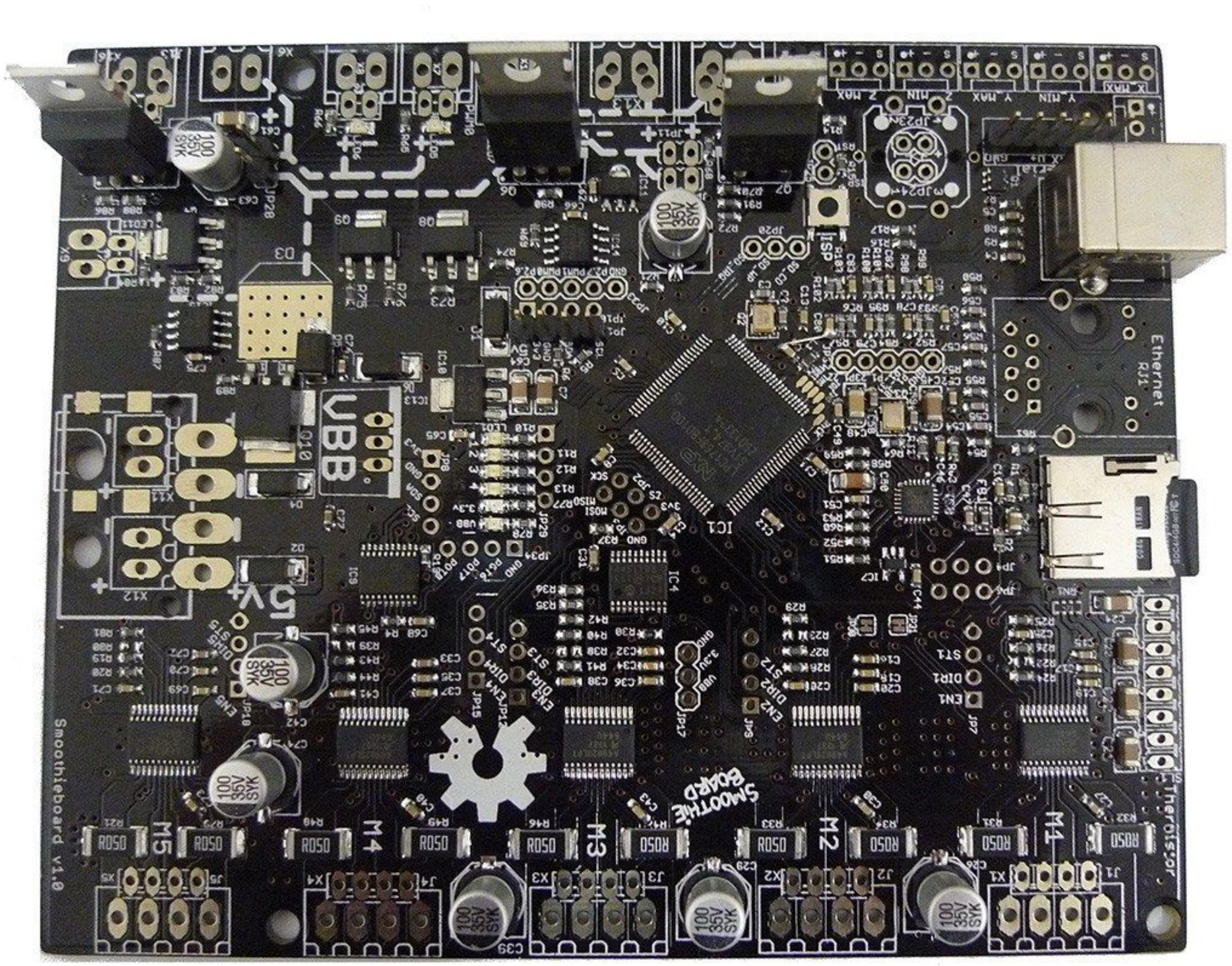

The 5X-xM version of the Smoothieboard does not have any of the connectors soldered in place. We take advantage of this and use header pins to connect an intermediate board (the Smoothieboard-Ribbon PCB). However, some of the connectors are used for MAPLE.

Install the connectors for M1, M2, X MIN and VBB (power in, the screw terminal receptacles). Also install the 5V switching regulator.

**Note**: do not connect or disconnect stepper motors while the Smoothie is powered on.

## Smoothieboard-Ribbon PCB

Solder the ribbon connector receptacle to larger board, as well as the four Schottky diodes (the white stripe of the diodes should be on the left with the board in the orientation shown). Solder the 1k (R1) and 2k (R2) resistors in place.

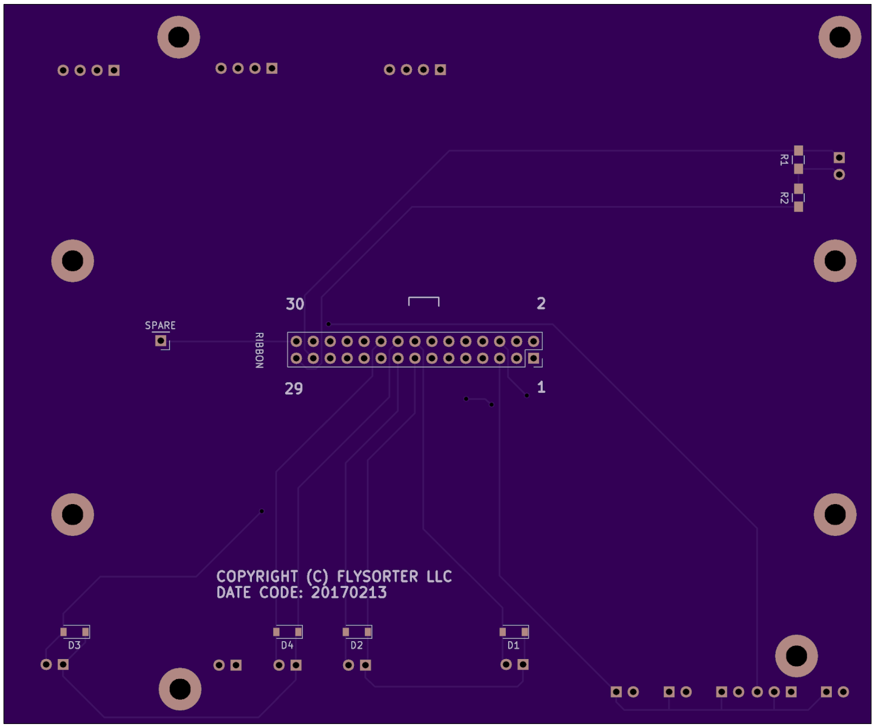


Then, insert the tall header pins between this board and the Smoothieboard and solder in place (the top side of the Smoothieboard should face out from the circuit board sandwich.

### Ribbon Breakout PCB

This PCB is mounted to the back side of the Z-plate. Before installing the board, solder another ribbon connector receptacle to this board, matching the orientation (pin 1) on the Smoothieboard-Ribbon PCB.

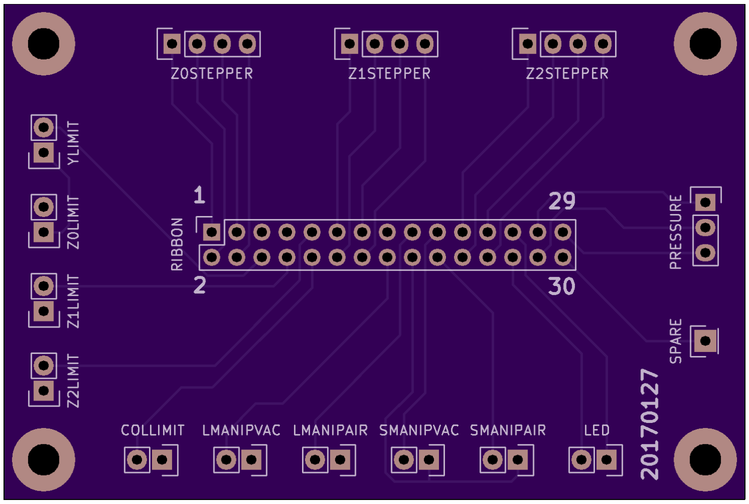


## Frame Assembly

First, assemble two identical rectangles that will form the sides of the frame with two of the 410 mm long 20x40 extrusions (the vertical members), one 1120 mm long 20x40 extrusion (the lower rail) and one 1120 mm long 40x40 extrusion with the linear rail glued to it, using four of the wide corner braces (HBLFSDK5). Be sure to slide the modified bearing blocks onto the rail before assembly, and be sure they are facing the same direction. Make sure the two OJUM-06-12 blocks with drilled & tapped M5

threads on the side are on the same rail, with the faces that you measured the 15 mm distance from on the inside. Use M5 x 10 mm socket head cap screws and extrusion nuts.

Use the notch in the laser-cut template to set the height from the top of the vertical rail to the top of the supported linear rail. Similarly, use the long section of the laser cut template to set the distance between the top and bottom horizontal rails.

Double check for square by measuring the diagonals with a ruler, tape measure, or string/wire.

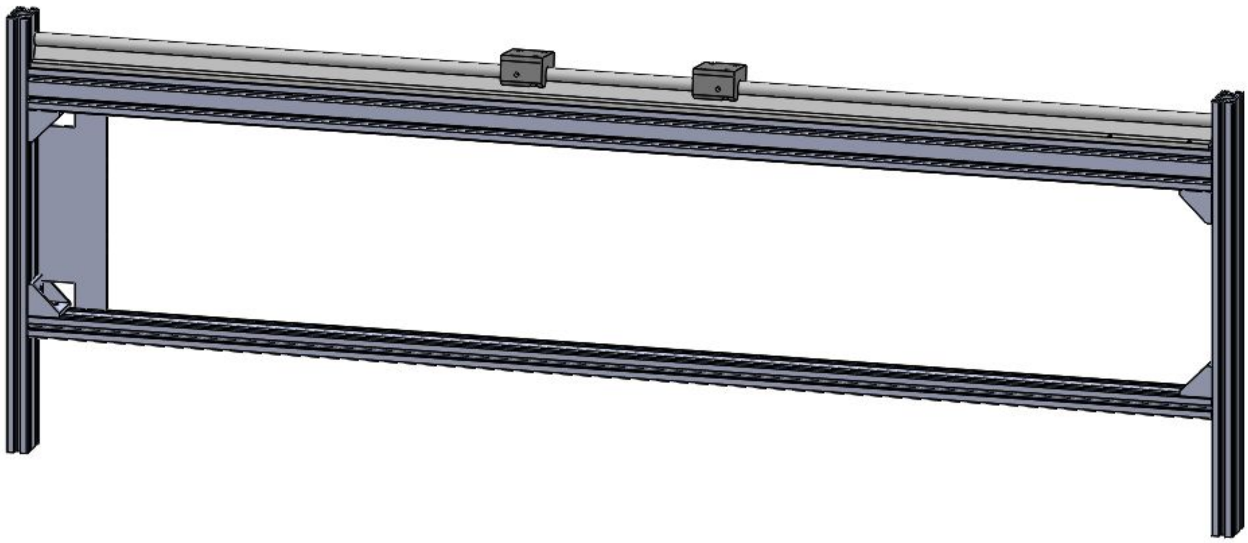

Next, use the triangular sheet metal corners (SHPTCUL5-SET) as well as the narrow corner braces (HBLFSSW5) to join the two sides. Lined up with the bottom rails are two 370 mm long 20x20 extrusions, and at the top are two 370 mm long 20x40 extrusions.

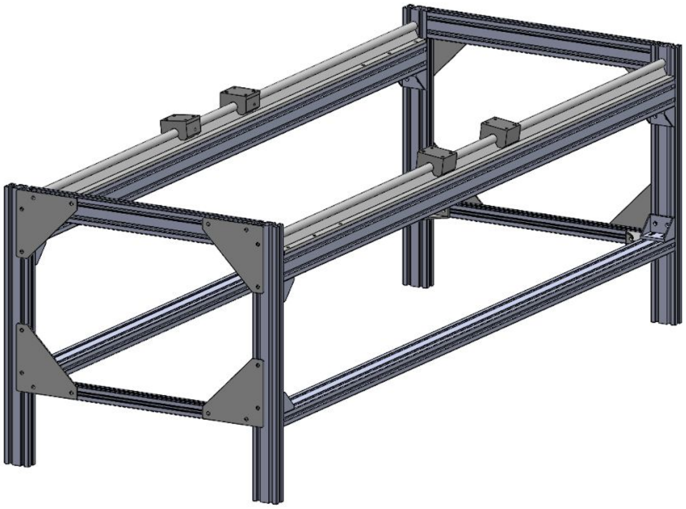

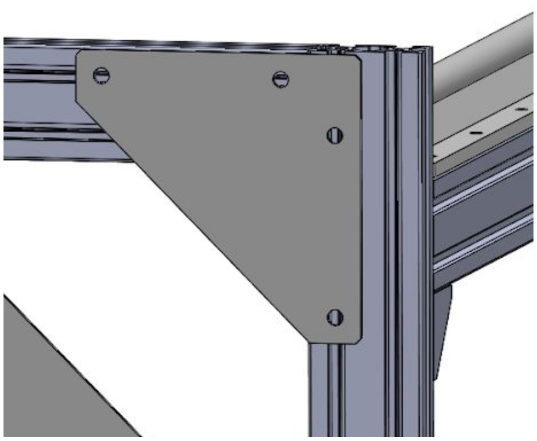

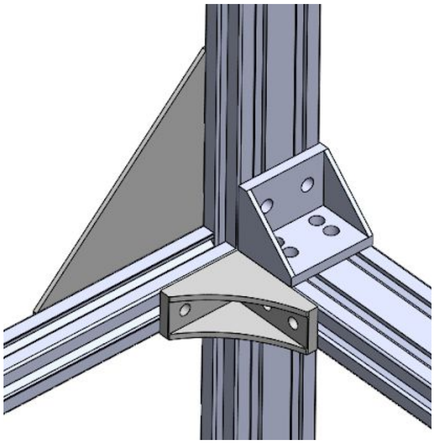

Again, check for square using a tape or string to measure across the diagonals (you can also use a large framing square).

Align the frame with the X axes left-to-right so the rail with the two OJUM-06-12 blocks with holes on the sides is in the back.

Attach the X axis limit switch holder to the rear right upright as shown, using M5 screws and insert nuts:

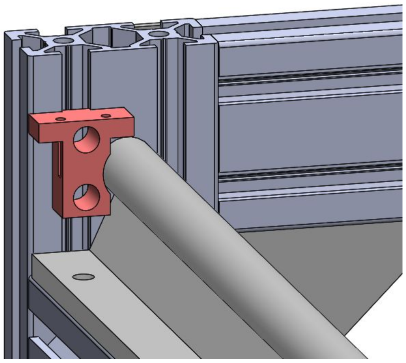

Attach the stepper plates to the top of the 410 mm vertical rails at the right end of the assembly. Attach the idler plates at the other end, with HBLFSSW5 brackets below each one. You will need to add Misumi post-assembly insert nuts on the top horizontal end rails (two per end, one for each plate).

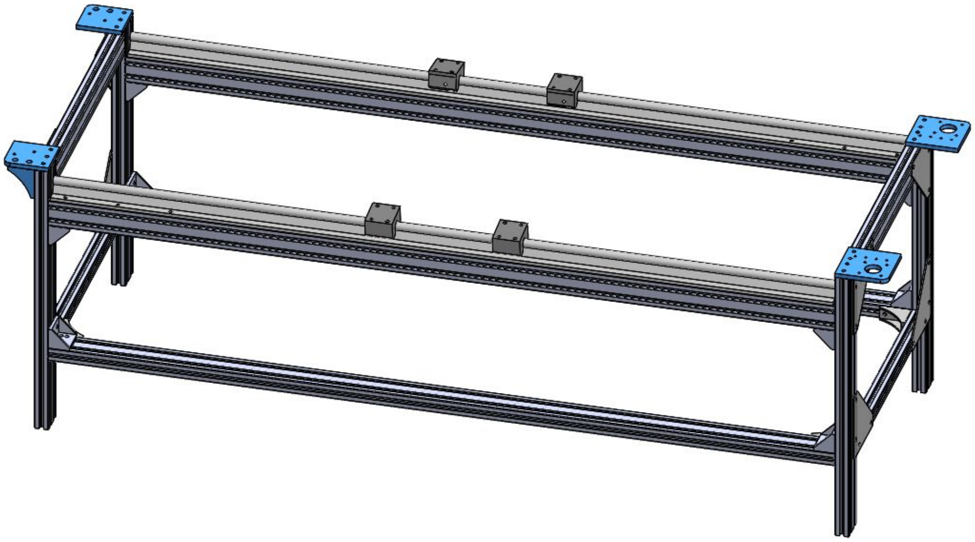

With the stepper motor plates on your right and the idler plates on your left, install the motors (using M3 bolts) and pulleys (using M5 shoulder bolts and 4 mm spacers on top) as shown:

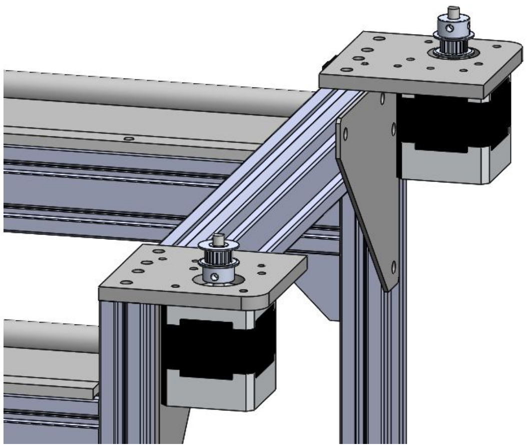

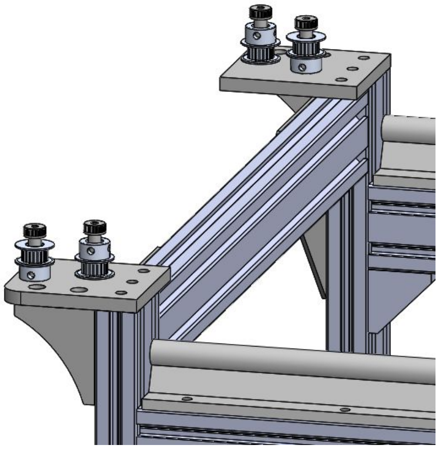

Using post-assembly insert nuts and M5 x 10mm screws, attach the EndPlate and WorkSurface to the frame as shown. Note that the WorkSurface sits underneath the side- and end rails, not on top.

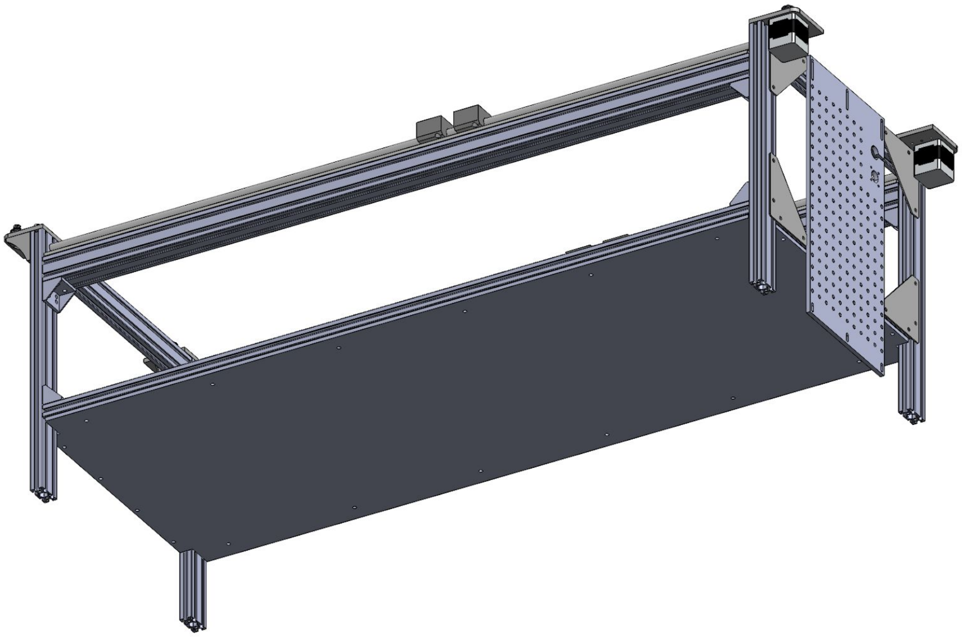

# Y Axis Assembly

Attach the upper and lower Y plates to the WJ200UM-01-10 bearings using M6 screws as shown:

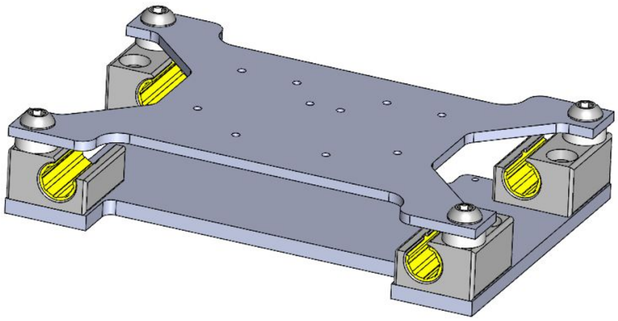

Then attach the two belt clamp parts in the orientation shown below (the two parts are distinct, and must be installed correctly for the belt heights to line up).

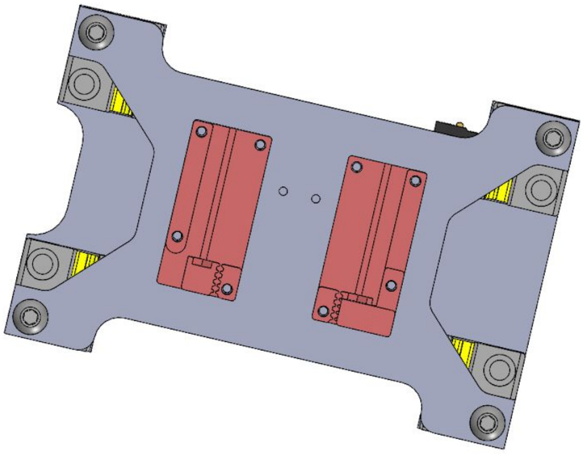

Slide the Y carriage assembly on to the WS-10-120 rail, then install the idler pulleys (above the ¼" nylon spacers) with M5 shoulder bolts. Note that if the screw threads from the shoulder bolts protrude below the bottom surface of the WS-10-120 rail, you'll need to cut / grind down the screws so they don't interfere with the X axis bearing blocks. Again, be careful to get the orientation of the carriage and the pulleys correct.

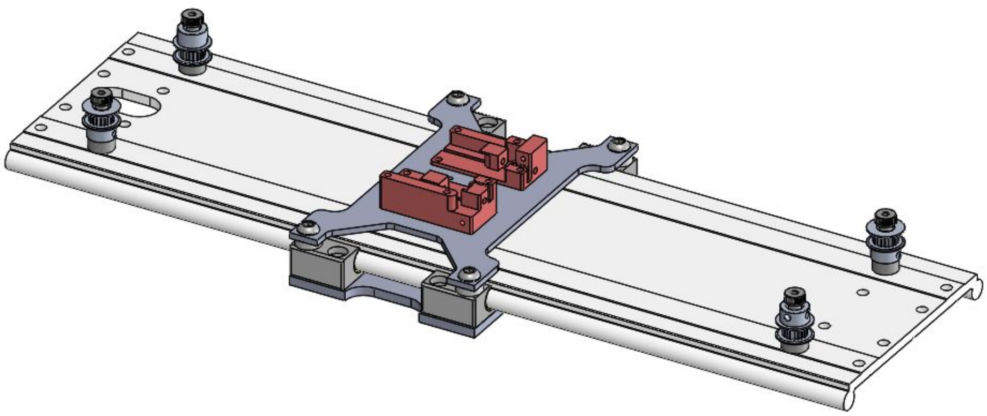

Attach the Y axis assembly to the X axis bearing blocks using M5 screws as shown. Each side requires 6 M5 screws, and the DragChain-YMount part is captured by two of them on the back rail. Do not tighten at this point.

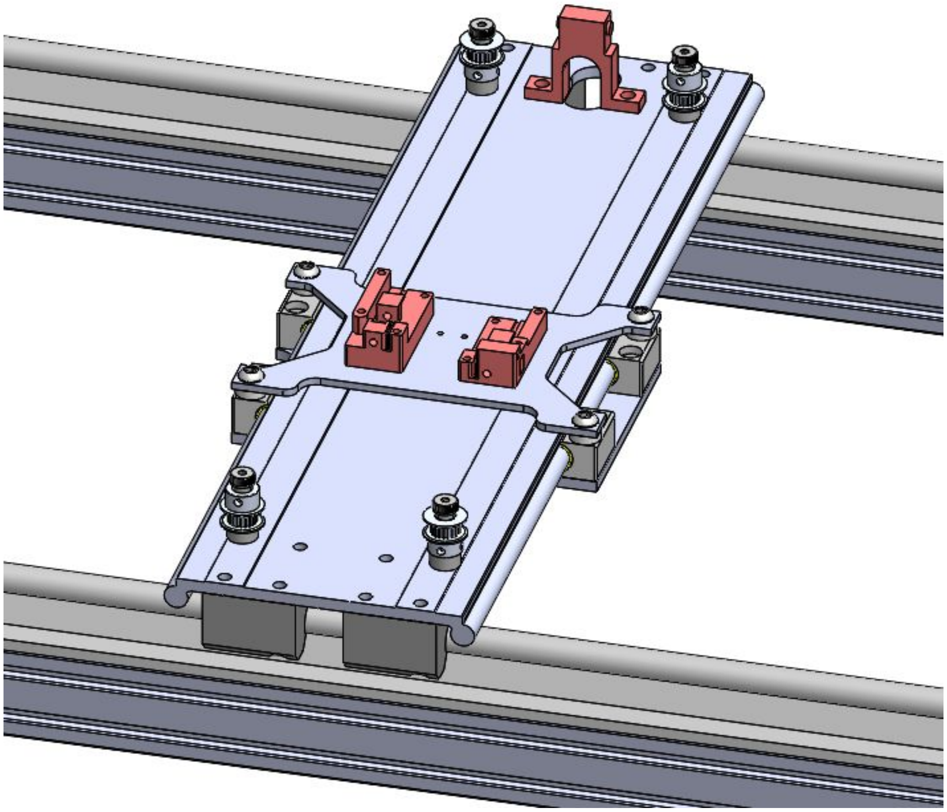

Finish assembling the belt clamps by inserting an M3 nut into each one, and then threading M3x25 screws through. Belt tension will keep the BeltAdjuster parts in place at the end of each screw.

Mount the DragChain-Plate between the two bearing blocks on the back rail:

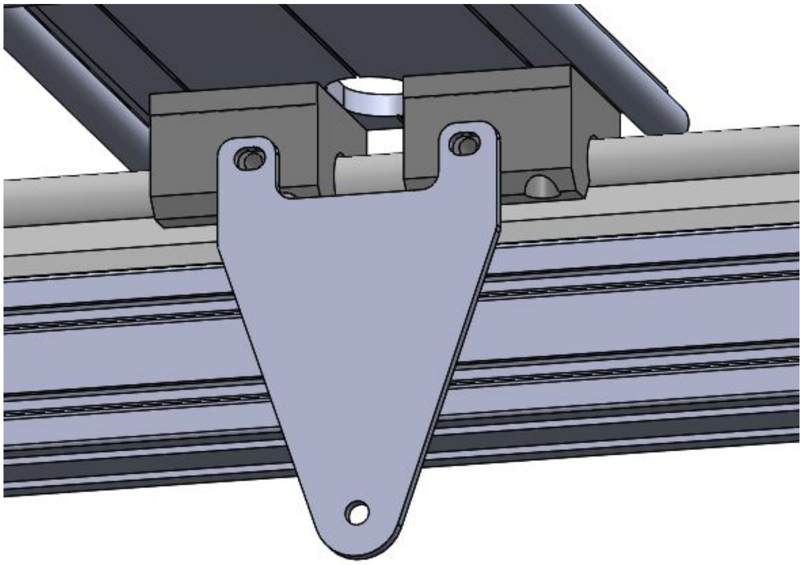

Cut two 3.5 m lengths of GT2 belt. Punch a small hole in one end of each belt, and use an M2.63 plastic screw to attach to the BeltClamps. Thread the belts as shown, snaking them through the belt clamps, and then using the slots in each clamp to hold the end of the belts. Trim excess.

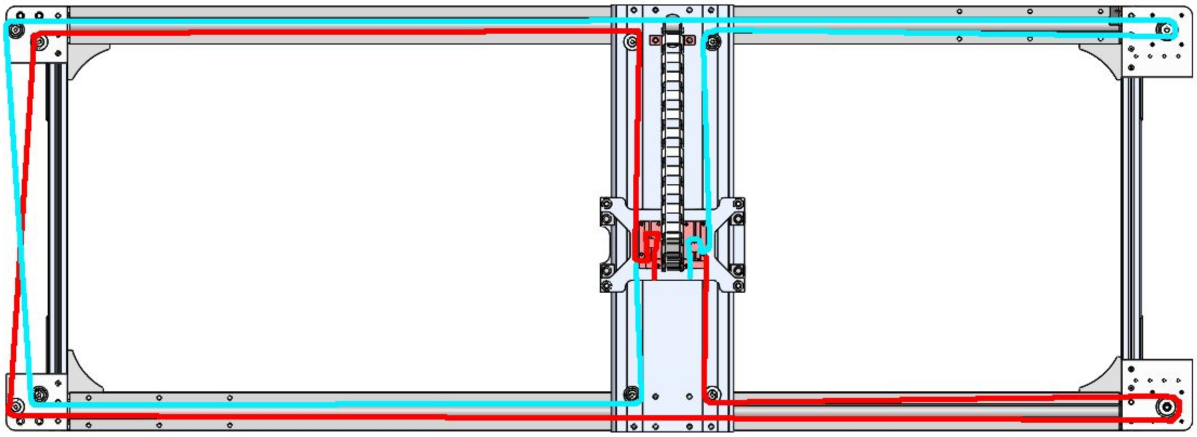

Belt tension and Y axis alignment will be addressed later on.

Attach the smaller drag chain between the Y carriage and DragChain-YMount, and the larger drag chain on the lower back rail, connecting to the newly installed DragChain-Plate with an M5 bolt and a nyloc nut.

## Rail Maintenance

Once the X and Y rails and bearings are assembled, they should be kept clean by periodically removing dust and debris from the rails using a dry cloth. Lubricant is not needed.

## Assembling the Z Axes

Attach the three IGUS SLN-27-14-0050-75-11-G-S-000 linear slides to the Z plate as shown, using 4 M2.5 x 10 mm screws for each slide.

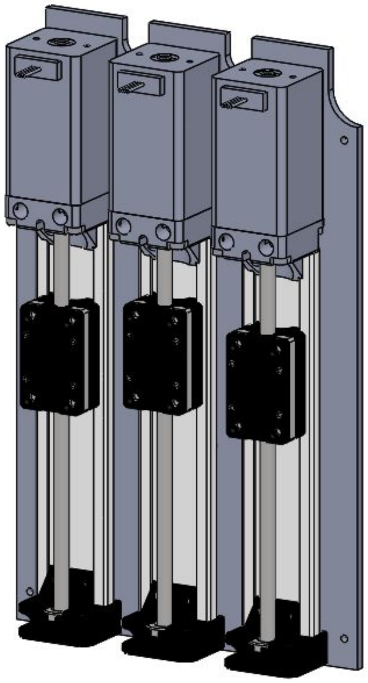

On the reverse side, attach the Clippard manifolds and valves as well as the Ribbon Breakout PCB (using M3 screws and 2 mm spacers). The PCB should be oriented with the stepper connections up.

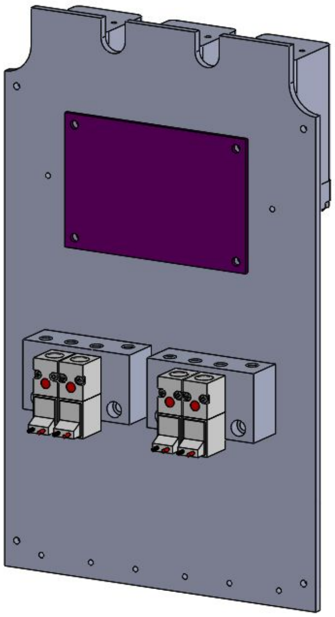

Then screw in 6 4 mm x M5 push-to-connect hose fittings to the Clippard manifolds.

## Z0 - Object Manipulator

Attach two 4 mm x M5 push-to-connect fittings to the top of the Object Manipulator Manifold, and the ⅛ NPT vacuum cup to the bottom. Attach the manifold block to a Z mount using M3 x 12 mm screws (you may need washers to keep the ends of the screws from protruding through the Z mount).

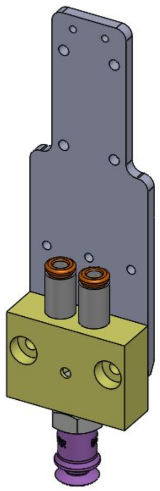

Attach the Z mount to the left-most Z slide using M3 x 10 screws.

## Z1 - Camera

Attach the lens holder to the camera PCB (you may need to remove the original lens holder). Then attach the camera PCB to the IS-Camera-Holder part using the M1.91 screws for plastics and the small, ¼” long nylon spacers to hold the board off the 3d printed part. Make sure the USB plug is in the orientation shown. Screw in the lens.

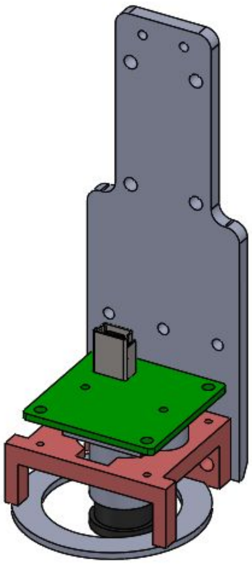

Attach the camera holder to the Z mount, then the assembled Z mount to the middle Z slide.

## Z2 - Organism Manipulator

Using diagonal cutters or a fine jewelers saw, carefully remove two 4 mm sections of the stainless steel tubing portion of the 0.042” OD and 0.028” OD dispensing needles. You can insert (steel) music wire of the appropriate diameters (0.032” and 0.019”, respectively) to prevent the tubing from collapsing during the cutting process. If needed, use a needle or diamond file to square up one end of each of the four tube sections, and align them so the clean ends face the same way. Insert these tubes as a group into the blunt needle (6710A61) as shown, and push them approximately 4 mm up, forming the fly aspirator:

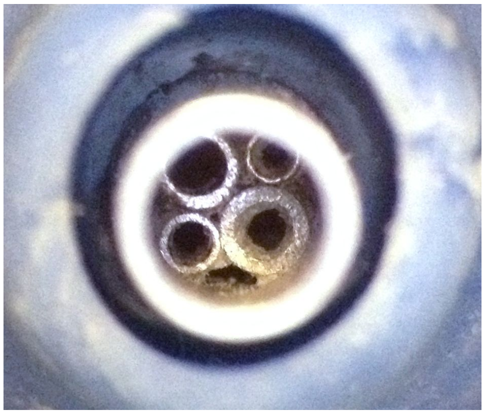

If the small tubes are too loose inside the blunt needle, you can use a little super glue or acrylic dissolved in a solvent (MEK or similar) as a bonding agent. The key is to use a thin liquid so the tubes remain open and air can flow through.

Attach two 4 mm x M5 push-to-connect fittings to the top of the organism manipulator manifold and a plastic Luer-lock quick turn coupling to the bottom.

Using M3 x 12 mm shoulder screws, attach the manifold to the remaining Z mount. The manifold assembly should rotate on the right-hand shoulder screw.

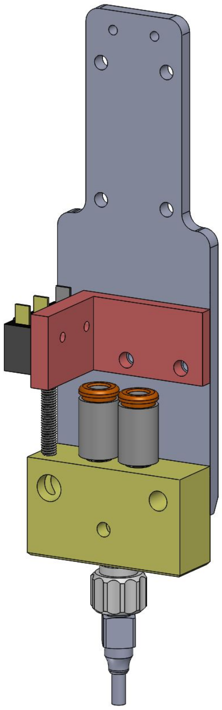

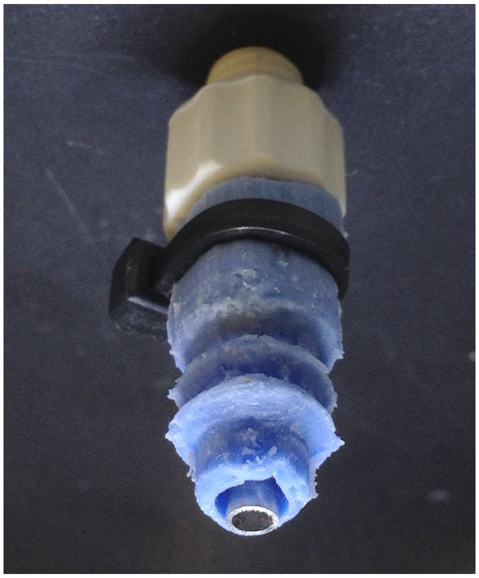

Screw in the 20 mm threaded rod to the top of manifold block, and add an M3 nut to lock in place.

Attach an SS-5 switch to the Z2 switch carrier using M2.5 screws, then attach the switch carrier to the Z mount as shown.

Adjust the M3 threaded rod up or down so that the switch is not triggered when the block is at its lowest point, but the switch triggers as soon as possible when you press up on the bottom of the block. Remove the switch (for now).

Attach the blunt needle (with tubes inserted) to the fitting on the bottom of the manifold block, then attach the silicone bellows with a small zip tie (see above).

Finally, attach the Z2 assembly to the third Z slide.

### Organism Manipulator Maintenance

With repeated use, organic residue will build up on the ends of the small inner tubes inside the blunt needle outer tube of the manipulator. This can be mechanically removed with a sharp needle (be careful not to dislodge the inner sections of tubing), or dissolved off with warm soapy water and gentle brushing.

## Complete Z Assembly

With the 3 end effectors mounted, attach the T brackets to the Z plate as shown using M3 screws:

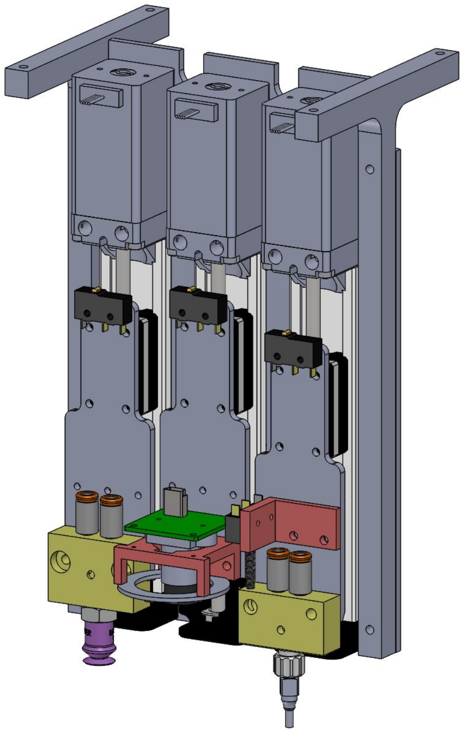

Then, attach the completed Z assembly to the underside of the lower Y plate. Ensure the 3 end effectors face forward (away from the larger drag chain)

# Wiring & Tubing

Unless otherwise noted, use 22 gauge stranded wire for all connections. It's helpful, but strictly speaking not necessary, to have several colors on hand to help differentiate between signals (i.e. power vs. ground).

For crimping, we like the [PA-20 universal crimp tool](). But in a pinch, careful use of pliers will do the trick.

All limit switches are wired between the common terminal (labeled 'C') and the normally closed terminal ('NC'). Thus, if they fail for some reason, the result is the same as the switch being depressed, and the software will not allow the robot to move.

## Threading the Drag Chains

It is easiest to thread wires and tubes through the drag chains several at a time (rather than all at once).

### Large Drag Chain

The large drag chain carries:

- 3x ~9' 10-pin ribbon cable (it's helpful to label them at each end)
- 1x 10' USB A-to-mini-B cable (the camera has a mini-B connector)
- 1x red (pink) 4 mm tube and 1x blue 4 mm tube (length depends on your air & vac connections)

Leave enough cable / tube on the entry end of the drag chain to connect to the Smoothieboard, air & vacuum outlets, and the PC (for the camera's USB cable).

### Small Drag Chain

The same cables & tubes exit the large drag chain, snake up through the slot in the Y axis, and then should be threaded through the small drag chain.

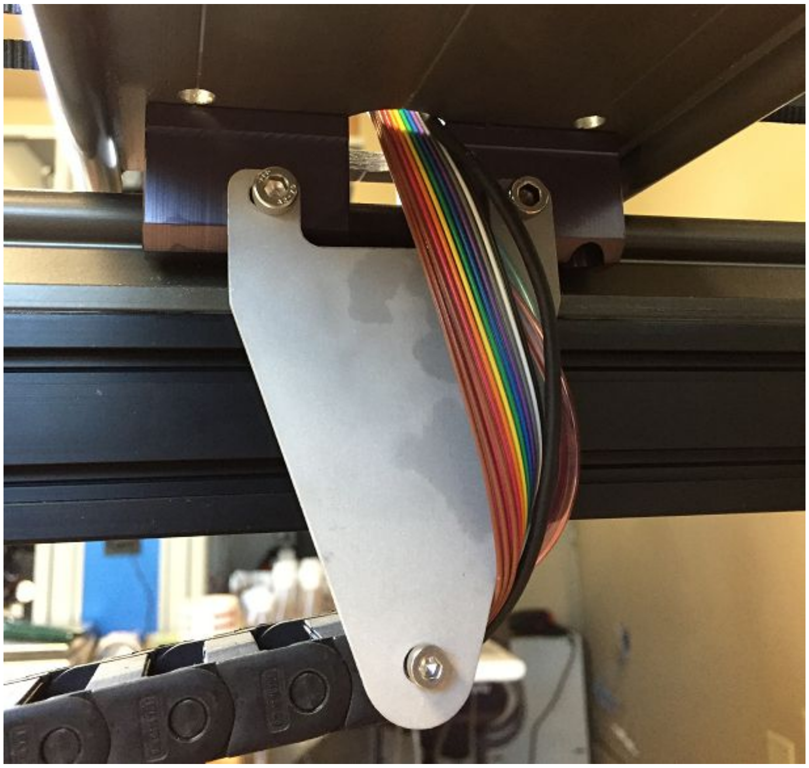

Connect the USB cable to the camera, and the pink and blue tubes to the inlets for the Clippard manifolds.

Use a vise or pliers to attach the ribbon cable connector plug to each end of the ribbon cables (it's a 30 pin connector and there are 3 10-pin cables). The order and orientation of the cables doesn't matter, as long as they are the same on each end.

The cables and tubes should pass to the left side of the Y carriage and around the back of the Z plate.

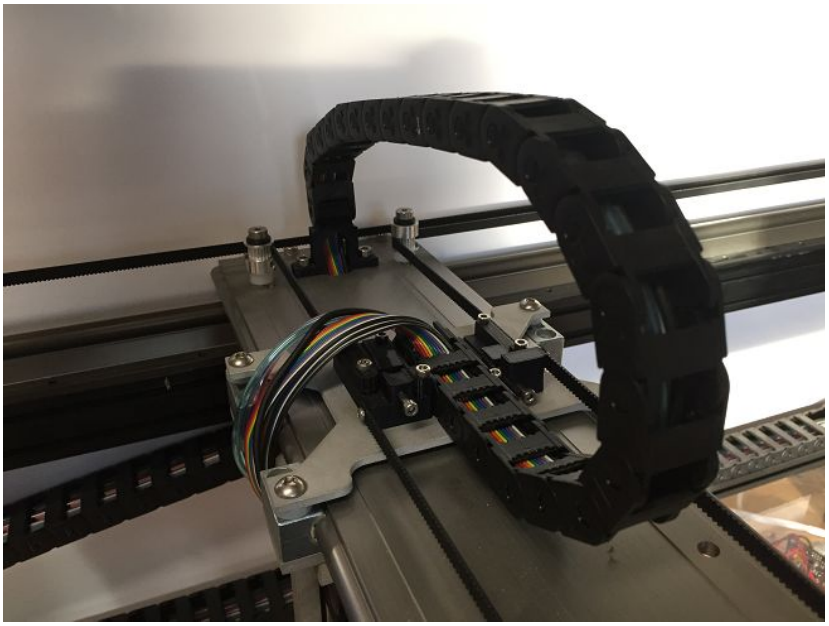

Wait to connect the ribbon cable to the PCB until after you've made all the wiring connections to that PCB.

## Power Supply

Connect the line, neutral & ground from the 120V cable to the appropriate terminals on the power supply. If desired, you can add the switch (DPDT) inline with the mains power.

Then connect wires (you should use something heavier than 22 gauge) to the +12 V and GND terminals on the power supply. The other end of those wires should go into the screw terminal block that mates with the power connector on the Smoothieboard for VBB. Be sure to check the polarity of that connector before powering the board.

## Smoothieboard

Solder approximately 1' of wire to the 'C' and 'NC' terminals of a limit switch, and crimp Molex pins on to the other end (the pins come with the Smoothieboard). Insert into a 3 pin housing as shown below.

Similarly, crimp pins on to the ends of the 4 wires from each CoreXY stepper, and insert into 4 pin housings.

These components connect directly to the Smoothieboard.

| Component | Smoothie Connection | Photograph |
|---|---|---|

| | **Point** | |
|---|---|---|
| X axis limit switch | X MIN header | 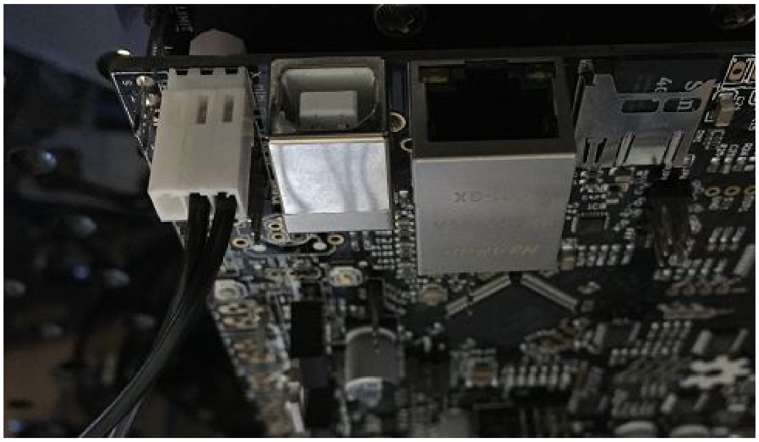 |
| Front CoreXY motor | M2 | 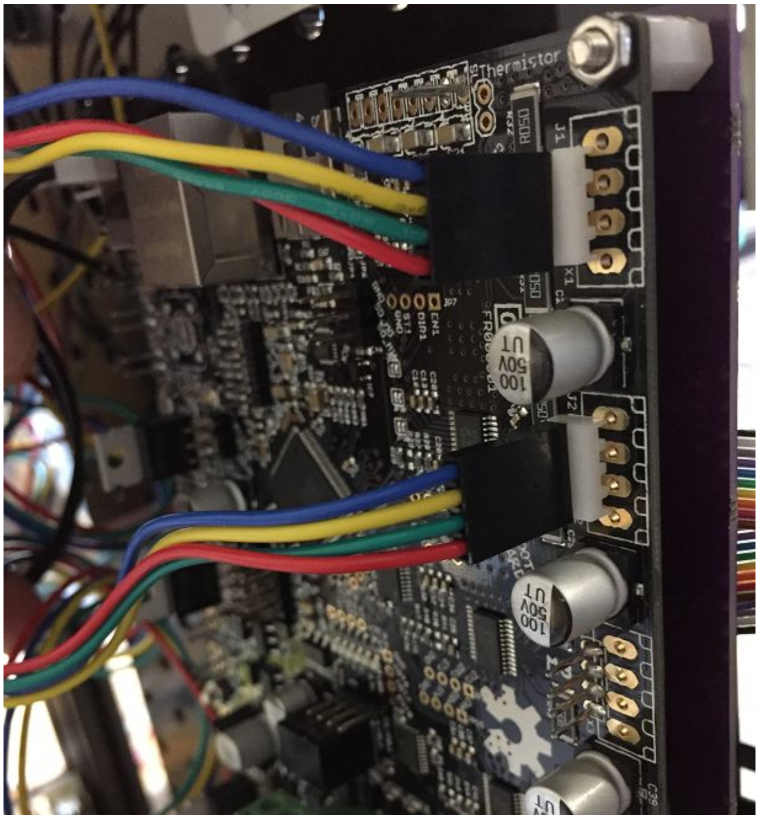 |
| Rear CoreXY motor | M1 | |

The rest connect to the Ribbon Breakout PCB on the back side of the Z plate.

## End Plate Assembly

The grid of holes on the end plate provides flexibility in mounting the power supply, Smoothieboard assembly, DPDT switch (in the round cutout near the top of the EndPlate), and any other components you may choose to include.

## Z Assembly

Before soldering to the Ribbon Cable Breakout PCB, prepare:

- 5x limit switches, soldering ~1’ lengths of wire to the C and NC terminals.

- The LED circuit board, with ~1’ lengths of wire soldered to the + and - terminals on the board (you may have to desolder the short lengths of wire that come on the board, or you can splice into them).
- The pressure sensor - solder ~1’ wires to pins 2 (red, 5V), 3 (black, GND), and 4 (blue, $V_{out}$)

Use epoxy or hot glue to affix the LED circuit board to the camera holder, run the wires up and thru the U channel to the back side. Solder the ground wire to the square LED pin and the positive wire to the round LED pin.

Similarly, affix the pressure sensor in the object manipulator manifold (the tube on the sensor fits inside the 3 mm hole in the manifold). Route the wires, then solder to the breakout PCB. The square pin is ground, next is +5 V, then $V_{out}$.

Run the wires from the 3 Z slide stepper motors through the U channels to the back side of the Z plate, and trim them before soldering to the breakout PCB. Solder the wires from the common (‘C’) terminals to the square pads on the board and the ‘NC’ terminals to the round pads.

Attach 3 of the limits switches to 3 Z mounts. You will have to bend the two outside terminals on the switches so they don’t collide with the screws that hold the Z mounts to the slides. Run the wires up and through the U channels, then solder to the breakout board as labeled.

Attach the 4th limit switch to the underside of the lower Y plate, and solder the wires to the appropriate pins on the breakout board.

Finally, attach the 5th limit switch to the Z2 switch carrier, route the wires, and solder to the PCB.

Solder the wires from the solenoid valves to the PCB. The black wires should go to the square pads on the board, and the red wires to the round pads. Keep track of which solenoid is connected to which set of pad (labels might help), so you can connect the correct tubes in the next step.

As shown in the image below, a zip tie anchor can help with cable routing.

Similarly, zip tie anchors can be attached to the front side of the 3 stepper motors at the top of each Z slide, and cables/tubes fixed in place. Be sure to leave enough slack in the cables so that the Z axes can move to their lower limits.

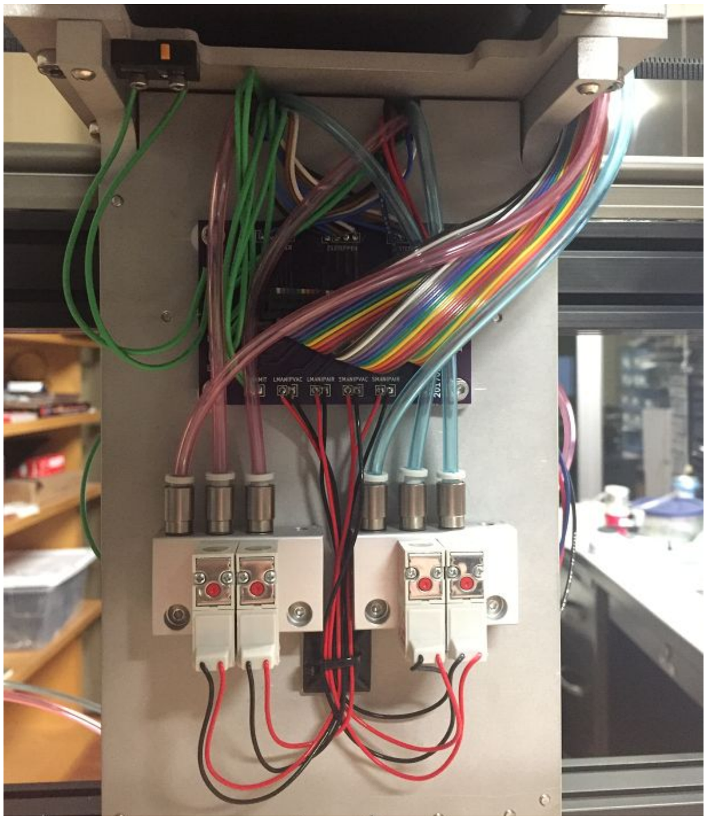

### Air & Vacuum Tubing

We use two different colors of tubing to differentiate positive and negative pressure. The red tubing specified in the BOM is actually a pink color, and we use the mnemonic “**P**ink is **P**ositive,” leaving the blue tubing for vacuum connections.

Run a short length of pink and of blue tubing through each U-shaped cutout at the top of the Z plate (where it meets the lower Y plate). On the front side, attach the tubes to the object and organism manipulators. On the back side, connect the two blue tubes to the vacuum manifold block and the pink tubes to the positive pressure manifold block.

## Firmware

Remove the microSD card from the Smoothieboard, mount it on your computer, and copy the *config* and *firmware.bin* files from the Smoothie subdirectory of the MAPLE Control Software github repository to the root directory of the card. Replace the SD card in the Smoothieboard, and when you next power the system, it will load the new firmware and use the updated config file.

## Alignment & Belt Tension

With the Smoothieboard not powered, unplug the CoreXY steppers from the Smoothieboard temporarily.

Use your hand to slide the Y carriage all the way to the right hand side of the robot. Tighten the 6x screws on each side of the Y carriage that fix it to the bearing blocks underneath.

Loosen the two M5 bolts that hold the front, top 40x40 aluminum extrusion to the right angle bracket on the left side of the machine. The slide the Y carriage all the way to the left side, and re-tighten the bolts.

Finally, tighten the M3 screws in the belt tensioner assemblies until the belts are quite snug. You aren't trying to deform any parts here, but the belts should make a low tone when plucked.

# Testing

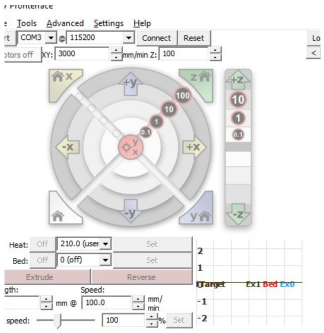


Download and install Pronterface - a program to send G-code commands to a COM port - [http://www.pronterface.com/](http://www.pronterface.com/)

Slide the carriage to the middle of the workspace. Connect the Smoothieboard to your PC with a USB cable, and power the Smoothieboard. Use the X & Y jog buttons to move the robot 1 or 10 mm in each direction. +X should move the carriage to the left, +Y should move it toward the front. If these directions are reversed, you may need to swap the M1 and M2 connectors, or reverse one or both of them (be sure to power down the Smoothie when making any changes).

Similarly, jog the Z axis up and down to make sure it goes in the right direction. +Z is towards the bed.

Once you're sure the robot is wired correctly, you can press the home button (or send the G28 command), which should zero the X, Y, Z (object manipulator), A (camera) and B (organism manipulator) axes in sequence.

You're now ready to download and install the MAPLE Control Software. Instructions and code available here: [https://github.com/FlySorterLLC/MAPLEControlSoftware](https://github.com/FlySorterLLC/MAPLEControlSoftware).
